# The Field Epidemiology Training Program’s Contribution to Essential Public Health Functions in Seven National Public Health Institutes

**DOI:** 10.3389/ijph.2023.1606191

**Published:** 2023-08-15

**Authors:** Angelina Cui, Sakina Hamdani, Mahlet A. Woldetsadik, Jacques W. Clerville, Audrey Hu, Aisha A. Abedi, Shelly Bratton, Reina M. Turcios-Ruiz

**Affiliations:** ^1^ Division of Global Health Protection, Center for Global Health, Centers for Disease Control and Prevention, Atlanta, GA, United States; ^2^ Oak Ridge Institute for Science and Education (ORISE), Oak Ridge Associated Universities, Oak Ridge, TN, United States; ^3^ Public Health PHI/CDC Global Health Fellowship Program, Public Health Institute, Oakland, CA, United States

**Keywords:** health systems strengthening, CDC, capacity building, field epidemiology training program, national public health institute

## Abstract

**Objective:** This study explores how Field Epidemiology Training Programs (FETP) whose National Public Health Institutes (NPHI) are supported by U.S. Centers for Disease Control and Prevention (CDC) have contributed to strengthening essential public health functions.

**Methods:** We conducted 96 semi-structured interviews with public health experts including NPHI staff, non-NPHI government staff, and staff from non-governmental and international organizations in Cambodia, Colombia, Liberia, Mozambique, Nigeria, Rwanda, and Zambia. We managed data using MAXQDA and employed direct content analysis to derive themes.

**Results:** Three overarching themes emerged in relation to FETPs’ role within the NPHIs’ public health functions. These themes included contribution to improving country surveillance systems, role in providing leadership in outbreak responses, and strengthening countries’ and the NPHIs’ surveillance workforce capacity. Participants also shared challenges around FETPs’ implementation and suggestions for improvement.

**Conclusion:** The results demonstrate the value of FETPs in strengthening public health systems through building workforce capacity and improving surveillance systems. By identifying the successes of FETPs in contributing to essential public health functions, our findings might inform current and future FETP implementation and its integration into NPHIs.

## Introduction

Field Epidemiology Training Programs (FETP) aim to produce a competent workforce of highly qualified field epidemiologists at all levels of the public health system to respond to public health threats in countries around the world [1]. FETP was modeled after the Epidemic Intelligence Service (EIS) [2], a 2-year applied epidemiology training program established by the U.S. Centers for Disease Control and Prevention (CDC) in 1951 [3]. The first FETP was established in Canada in 1975 and there are now programs in more than 60 countries [4]. Since 1980, CDC has supported the establishment of FETPs around the world [5], which aligns with the International Health Regulations (IHR) (2005) that mandate World Health Organization (WHO) member states should build capacities to “detect, assess, notify, and respond” to potential public health emergencies [6].

The FETP model focuses on building capacity in key public health competencies and comprises of three training tiers that can be tailored to meet specific needs of countries or regions: Frontline, Intermediate, and Advanced [4]. These public health functions include activities such as epidemiologic surveillance of diseases, outbreak investigation and response, evidence-based decision-making, and scientific communication. Some FETPs have added a laboratory component, Field Epidemiology Laboratory Training Programs, to strengthen laboratory capacity. In addition, human resources development is one of the core public health capacities required for effective implementation of the IHR (2005) [7]. Building capacity in key public health functions is imperative in our highly interconnected world where public health threats are increasing. The current COVID-19 pandemic is a concrete example that underscores the need for adequate human resources globally for key public health response activities [8]. FETPs have been instrumental in supporting global COVID-19 preparedness and response efforts in a variety of areas such as screening at points of entry, infection prevention and control, risk communication, case investigation and management, and coordination [9]. For example, during COVID-19, FETP graduates in Uganda trained village health teams to build up community-level response capacity, conducted multiple outbreak investigations in prisons, and presented real-time COVID-19 data, which led to immediate data driven decision making [10,11].

In addition to their role in conducting critical public health functions, FETP graduates are expected to occupy leadership positions, mentor FETP residents [4], and work closely with partners and other public health experts within national public health institutes (NPHIs) and ministries of health [12]. For instance, in Zambia, FETP graduates are integrated into the Emergency Operations Center (EOC), which is embedded in the NPHI, and are established as essential members of outbreak response teams. They continue to participate in many infectious disease outbreak investigations, publish, and present their findings at national and international scientific conferences. Their presence has led to more thorough and regular outbreak investigations in Zambia. In Nigeria, FETP residents and graduates composed a multi-sectoral and multi-disciplinary team that works together to provide a range of public health services, including carrying out epidemiological studies [13]. Lastly, FETP graduates have led rapid response teams and trained health workers on Ebola infection control and prevention to strengthen in-country capacity to prepare for potential Ebola Virus Disease (EVD) outbreaks in the future [14]. These examples underscore the versatility of FETPs to undertake multiple functions in different settings, at any levels of their public health system. Therefore, well-functioning FETPs are expected to strengthen capacities of NPHIs, which ensure coordination of disease surveillance, outbreak investigation, and public health emergency management.

As of March 2021, 86 FETPs have been established, providing access to training in field epidemiology to persons from more than 165 countries and territories [15]. While CDC acknowledges the contribution of FETPs in strengthening health systems in various settings, the perceptions of stakeholders in countries with established FETPs have yet to be well-documented. In 2019, CDC conducted an evaluation to assess the impact of its investment in developing and strengthening NPHIs in seven countries. This paper is focused on presenting themes that emerged around how FETPs contributed to essential public health functions within the seven NPHIs included in this evaluation.

## Methods

We employed an exploratory qualitative study design and applied purposive sampling to select participants from seven countries where CDC supported NPHI development and strengthening. These countries were Cambodia, Colombia, Liberia, Mozambique, Nigeria, Rwanda, and Zambia. We conducted semi-structured interviews with 96 stakeholders (range 10–19 stakeholders per country), including NPHI staff (e.g., NPHI director, center leader), non-NPHI government staff (e.g., Ministry of Health (MoH) officials, district health leaders), and partners that work closely with NPHIs (e.g., international organizations, non-governmental organizations). A detailed list of participants’ institutions and their positions is presented elsewhere [16]. Interviews were conducted in-person (except for two interviews that were conducted via telephone) from August 2019 through January 2020 by MAW, who is a PhD researcher and was the lead of the evaluation, and a fellow who had experience working in sub-Saharan Africa. MAW provided training on qualitative methods to team members who participated in data collection, analysis, and writing. The interviews lasted 41–96 min (average = 53 min) and were audio-recorded if the participant agreed (two participants declined to be audio-recorded). All interviews were conducted in English, except 13 interviews in Colombia, and one interview in Cambodia, which were conducted through experienced interpreters in Spanish and Khmer, respectively.

All recordings were transcribed verbatim and coded using MAXQDA Version 20.0.2 by AC, SH, and MAW. The data were analyzed using a directed content analysis approach, which began with the interview questions as a guide for developing initial themes, while also allowing for flexibility so that additional themes could emerge directly from the data [17]. The analytic team reviewed a random selection of coded transcripts to ensure consistent application of theme categorizations and used an iterative process to resolve any discrepancies in the coding application. This process established inter-coder reliability, including consistency and consensus coding application within the MAXQDA platform. We shared our findings and conclusions with a small group of representatives at each of the seven NPHIs to assess and refine the validity of our conclusions. In addition, to reduce the potential for evaluator bias during data analysis, the team regularly debriefed to discuss emerging themes and conclusions [18]. This evaluation was reviewed by CDC’s project determination process and the Office of Management and Budget was exempted and did not require review from institutional review boards. Participants were notified of data confidentiality, data safeguarding procedures, and their rights regarding participation. All participants provided written consent before the interview.

## Results

A total of 96 stakeholders from Cambodia, Colombia, Liberia, Mozambique, Nigeria, Rwanda, and Zambia participated in the interviews. Forty-three (45%) were NPHI staff, 29 (30%) were non-NPHI government staff, and 24 (25%) were staff from non-governmental and international organizations working in the seven countries. Participants provided general impressions about the program and identified numerous roles FETPs play in enhancing public health functions within NPHIs. These included: (1) FETPs’ role in improving surveillance systems, (2) FETPs’ role in providing leadership during outbreak response, and (3) FETPs’ role in strengthening NPHIs’ workforce capacity. Participants also identified challenges with FETPs and suggested a few areas for improvement.

### Participants’ Perspectives of FETPs in General

Participants viewed FETP as an integrated program that played an essential role in supporting public health functions within NPHIs. When stakeholders discussed their perspectives of FETPs, they highlighted that FETPs served as an essential component of NPHIs, and that FETP was critical for the career development of surveillance officers. One participant shared:

“FETP is a crucial area of the INS [Institute of Nacional de Salud], especially with pandemic preparation work and in other areas of [public health] cooperation” [Colombia (4), INS]

Participants who had graduated from FETP shared personal accounts of how the program was instrumental in preparing them for their career development and current work. They also shared how they have seen transformative results in skills learned from colleagues who have gone through FETP. Two participants gave specific examples:

“From where I stand and the interaction that I’ve had with a number of colleagues that have gone through the program [FETP], I see it being transformative.” [Zambia (9), Ministry of Health (MoH)]

### FETPs’ Role in Improving Surveillance Systems

Participants cited how FETPs played an important role in improving surveillance systems by improving surveillance officers’ ability to detect diseases and investigate outbreaks.

“It’s clear when you look at the skills sets of people, what they are able to get out of it [FETP], you really see a very clear difference between what they were before and what they become afterwards, in terms of skills in surveillance, use of health data to inform decisions, and then so on.” [Zambia (9), MoH]

Participants shared how surveillance officers trained in the FETP were able to detect diseases effectively and efficiently. In addition, trained surveillance officers exhibited improved skills in data collection and management, data presentation, and evidence-based decision making. These skills contributed to FETP surveillance officers’ ability to identify and respond to outbreaks.

“I think one area where they [FETP graduates] have supported us a lot is with respect to detection. When I say detection, I’m talking about improving surveillance, because the US CDC has helped us to—to train our frontline healthcare workers and surveillance officers in field epidemiology, which is very fantastic.” [Liberia (11), National Public Health Institute of Liberia (NPHIL)]

FETP graduates also improved documentation of outbreak investigations through completion of after-action reviews and disease analysis. Participants concluded that FETPs’ ability to improve surveillance officers’ disease detection and outbreak investigation skills contributed to an overall improvement in surveillance systems.

“During the cyclone they [FETP trainees] were very active and were involved in the investigation of outbreaks… INS (Instituto Nacional de Saude) helped us make informed decision based on evidence.” [Mozambique (12), Department of Public Health at Provincial Health Directorate (PHD)]

### FETPs’ Role in Providing Leadership During Outbreak Response

Many participants mentioned how FETP graduates served in pivotal leadership roles in outbreak investigations. FETP graduates were on the forefront of various outbreak investigations such as pandemic influenza in Colombia and cholera in Zambia. According to participants, FETP equips surveillance officers with the skills and confidence to work independently in initiating outbreak investigations–collecting information, analyzing data, and communicating with stakeholders–resulting in FETP graduates serving as leaders during outbreak investigations. One participant stated:

“Whenever there is an outbreak, [FETP graduates] can just make a descent on [the] field and start collecting samples, collecting information, analyzing them, and communicate to people in the right way…” [Rwanda (6), Rwanda Biomedical Center (RBC)]

Participants mentioned how FETP graduates hold positions at high levels of both government and non-governmental organizations, as well as throughout all levels of government. FETP graduates at both district and national levels of government identified outbreaks early and improved outbreak reporting. These skills in early identification and reporting have created stronger feedback mechanisms between district and national levels. Efficient reporting has enabled national levels to provide better support to lower levels, which did not happen before.

“We had surveillance officers at the national level, at the county, at the district, even at some health facility levels, who have been trained in frontline FETP and intermediate FETP. And that has helped them a lot, because they are now able to identify outbreaks early, which is very good, and they are able to report the outbreaks to the national level and they are able to respond with some support from the national level, which did not happen in the past” [Liberia (11), NPHIL]

### FETPs’ Role in Strengthening the NPHI’s Workforce Capacity

Participants described Frontline, Intermediate, and Advanced tiers of FETP as vital parts of their respective country’s NPHIs. Training in field epidemiology enhanced their ability to respond to public health emergencies by providing a workforce that is trained through an EIS-modeled approach, as well as increasing lab capacity and necessary technology in the country. This workforce can be mobilized quickly at the national and subnational levels. Participants cited that FETP Frontline training of surveillance officers at district and sub-district levels have increased the capacity of available workforce at the district level and in areas that were once underserved, so that they can now respond to local outbreaks without relying on a national response.

“It is difficult to talk about Zambia National Public Health Institute (ZNPHI) without talking about the FETP. We see it embedded in the institution – I think that it should really remain one of the flagship activities of ZNPHI.” [Zambia (9), MoH]

Additionally, skills such as data analysis and writing were also mentioned as having been improved through FETP trainings which was cited as a reason FETP graduates were able to attain leadership positions within Ministries of Health.

“When we look at the data analysis, people at central level but also at district and health center level, have been trained on surveillance of data and to look at the data and maybe also developing policy brief, developing scientific papers. This was done and even developing grants, we were not able to develop in the grant writing, it was a very big gap in the grant writing.” [Rwanda (10), RBC]

### Challenges With FETPs

When discussing the way forward for their institutions, participants raised challenges centering on two themes: 1) Challenges with academic certification of FETP and 2) Challenges with formalizing FETP trainings.

Most of the participants stated they believed formalizing FETP in their country was the next step. Participants mentioned how if FETP is not a degree program recognized by their Ministry of Education, that despite being internationally accredited, the program is less attractive and creates a barrier for FETP trainees’ professional advancement. Participants expressed desire for their national public health institutes to become licensed as educational institutes to add “weight” to their FETP training and allow them to provide degrees without depending on their Ministries of Education.

“Because the design of the program from the very beginning, even at the recruitment process, it’s not the academic system. So, we cannot integrate into our academic system because those programs have been designed and implemented for non-academic purposes. So right now, there’s just some thinking about how to become an academic program, but there is a mismatch in the requirements, so if they want to become an academic program, there needs to be a major restructuring”. [Cambodia (1), Cambodia National Institute of Public Health (NIPH)]

All participants emphasized the need to institutionalize FETP within their NPHI or in collaboration with a local university. Others described a relationship in which FETP is housed in the national public health institute and local medical universities which provide degrees to the trainees. However, it was also stated that converting FETP into an academic program would pose a challenge due to the way FETPs are designed and implemented in the surveillance system, for non-academic purposes. Therefore, this would require a major restructuring of the program.

“…the design of the program from the very beginning, even at the recruitment process, it’s not the academic system. So, we cannot integrate into our academic system because those programs have been designed and implemented for non-academic purposes.” [Cambodia (1), NIPH]

### Suggested Improvements for FETPs

While discussing the future of FETP, participants pointed out areas of improvement. Two themes that emerged were 1) professional development for trainees and 2) leadership and management. Many participants cited lack of job prospects and low salary as hindrances to recruitment. Lack of political will to fund public health, in general, and public health workforce development, specifically, was cited as a cause of this issue. Many field epidemiologists must serve in remote areas under difficult work settings and participants felt that this is not sensible to the average person to train in the equivalent of a master’s degree but earn only minimum wage at the end of it.

“Field epidemiologist who work in most areas work under difficult and strenuous conditions with a low salary…what is a reasonable salary for a field epidemiologist who went to various trainings and is now working in a remote area? It has to be worthwhile for them to do this work after going through all that training.” [Cambodia (12), German Development Agency (GIZ)]

Another participant expressed that the program needed to improve leadership and management to make the FETP’s work more visible within the country. Participants from Colombia expressed that having teams in every area led to some of them being underutilized and are working to improve their approach to conveying risk to the community. Part of this effort is strengthening leadership and management both leadership and management content of the FETP curriculum as well as leadership and management of staff implementing FETP.

“We’ve been strengthening our efforts specially in terms of our risk communication strategy. A lot of communication activities came out from the maturity framework exercise, and I think that those are also important to strengthen. One of these activities include leadership and management. The institute should strengthen its leadership and management efforts internally as well as externally.” [Colombia, (2), INS]

## Discussion

Throughout this study, participants commented on FETP’s role and significance in supporting NPHI’s public health functions, and the importance of having FETP integrated into NPHIs. Our results highlight and provide evidence that FETP improves country’s surveillance systems, provides leadership during outbreak responses, and strengths workforce capacity within NPHIs. Most crucially, participants expressed the program’s ability to improve surveillance officers’ skills in outbreak investigation and disease detection. Participants further shared how FETP graduates exhibited improved skills in reporting and investigating outbreaks, collecting and managing data, and making evidence-based decisions. During the COVID-19 pandemic, we saw evidence where FETP graduates executed these skills in various countries, by staffing emergency operation centers, building out community-level health surveillance capacity, and taking on leadership roles at national levels and during outbreak investigations [9,10].

In addition, participants noted that FETP has strengthened workforce capacity, by increasing the availability of trained surveillance officers at the district and sub-district level. Participants mentioned how increased capacity of trained surveillance officers allows the district level to respond to public health emergencies in a more timely and effective manner, without having to rely on national surveillance teams.

Participates reported on challenges in FETP implementation, such as accreditation and institutionalization, and identified strategies for improvement. Participants urged that FETP become associated with some form of academic certification but noted that barriers lie within existing processes of government and academic institutions. Participants continued to suggest increasing opportunities for professional development and improving leadership and management components in FETP curriculum.

These findings align with challenges and suggestions mentioned in previous FETP evaluations and existing literature. There are noted challenges in how FETP are linked with universities, ministries, and international agencies, and in the process of how FETP becomes institutionalized [19,20]. FETP graduates in Ethiopia and Tanzania also suggested to provide refresher courses and additional training, as well as to improve mentorship [21,22].

Our findings indicate that FETP enhances public health functions within NPHIs and contributes to stronger public health systems. It will be important to consider challenges in FETP implementation, in order to increase success and utility of the program. Ownership of FETP by an NPHI or Ministry of Health is an essential element for FETP institutionalization and sustainability [20]. By further investigating how to address FETP challenges, NPHIs and countries will be able to enable FETPs to maximize impact on strengthening health systems at all levels.

There are limitations that should be considered when discussing our findings. The interview questions may not have accurately reflected the experience of countries that have FETP but not NPHI. We believe our findings offer valuable information on the function of FETP within NPHI in enhancing public health functions. In addition, data were only collected from seven countries [16]. Countries with NPHIs were selectively and purposefully sampled and sampling did not take into consideration countries with FETPs.

### Conclusion

There is evidence to show that Field Epidemiology Training Programs are impactful programs that provide value within countries’ NPHIs and help strengthen global health protection and health systems, which supports the vision set forth in the recently developed Global Field Epidemiology Roadmap [23]. FETPs have been shown to improve surveillance officers’ skills, from identification and reporting of outbreaks to timely investigations, communication between levels of government, and overall strengthen the country’s surveillance systems. FETP also serves as a valuable program in training surveillance officers and building workforce capacity, thus offering a solution to the gap of workforce development in these countries. It is important to consider our findings as evidence that FETPs should be integrated into NPHIs and highlights the success of FETP as a mechanism for strengthening essential public health functions in countries.
